# Роль конечных продуктов гликирования в развитии и прогрессировании диабетической нейроостеоартропатии

**DOI:** 10.14341/probl12778

**Published:** 2021-07-04

**Authors:** М. М. Каландия, А. Ю. Токмакова, Г. Р. Галстян

**Affiliations:** Национальный медицинский исследовательский центр эндокринологии; Национальный медицинский исследовательский центр эндокринологии; Национальный медицинский исследовательский центр эндокринологии

**Keywords:** сахарный диабет, стопа Шарко, ДНОАП, остеоартропатия, конечные продукты гликирования

## Abstract

Диабетическая нейроостеоартропатия (ДНОАП, стопа Шарко) является серьезным осложнением сахарного диабета, генез которого не окончательно изучен. Данная патология в большинстве случаев поздно диагностируется, что приводит к развитию тяжелых деформаций стопы вплоть до потери опороспособности конечности. Не существует единственной гипотезы формирования стопы Шарко, но есть факторы, предрасполагающие к ее развитию, а также ряд вероятных провоцирующих событий. Избыточное образование и накопление конечных продуктов гликирования (КПГ) может играть важную роль в патогенезе этого осложнения СД. КПГ — различные соединения, образующиеся в результате реакции между углеводами и свободными аминогруппами белков, липидов и нуклеиновых кислот без участия ферментов. Существуют различные факторы, которые приводят к накоплению КПГ в организме человека. Выделяют эндогенные и экзогенные факторы. К первым относятся определенные заболевания, такие как сахарный диабет, почечная недостаточность, ускоряющие процессы гликирования. К экзогенным факторам, приводящим к образованию продуктов липоокисления и гликоокисления, относятся табачный дым и длительная термическая обработка пищи.

В данном обзоре представлена информация о роли КПГ в развитии и прогрессировании осложнений у пациентов с сахарным диабетом.

## Abstract (en)

Diabetic neuroarthropathy (DNOAP, Charcot’s foot) is a serious complication of diabetes mellitus, the genesis of which is not fully understood. In most cases, this pathology is diagnosed late, which leads to the development of severe deformities of the foot, up to the loss of support ability of the limb. There is no single hypothesis for the formation of Charcot’s foot, but there are factors predisposing to its development, as well as a few likely provoking events. Excessive formation and accumulation of end products of glycation may play an important role in the pathogenesis of this complication of diabetes. End products of glycation (AGE) are a variety of compounds formed as a result of a non-enzymatic reaction between carbohydrates and free amino groups of proteins, lipids and nucleic acids. There are various factors that lead to the accumulation of AGE in the human body. Allocate endogenous and exogenous factors. The former include certain diseases, such as diabetes mellitus, renal failure, which accelerate glycation processes. Exogenous factors leading to the formation of lipo-oxidation and glyco-oxidation products include tobacco smoke and prolonged heat treatment of food.

This review provides information on the role of glycation end products in the development and progression of complications in patients with diabetes mellitus.

Сахарный диабет (СД) — одно из самых распространенных хронических заболеваний в современном мире, которое достигает масштабов эпидемии. По данным IDF (International Diabetes Federation), им страдают 463 млн человек, а к 2030 г. прогнозируется увеличение числа больных до 578 млн [1]. Прогресс медицинской науки в целом и эндокринологии в частности ведет к увеличению продолжительности жизни пациентов с СД, что, в свою очередь, приводит к росту числа лиц с выраженными микро- и макрососудистыми осложнениями данного заболевания [2].

Наиболее частым из них признается нейропатия, которой, по некоторым данным, страдают до 60% пациентов с диабетом [3]. В последнее время появляется все больше данных о распространенности нейропатии у лиц с предиабетом (нарушением гликемии натощак или нарушением толерантности к глюкозе) [4][5]. В работе, проведенной Yi-ChenLin, было выявлено, что изменения в аксонах начинаются у пациентов еще на стадии предиабета. Следовательно, скрининг полинейропатии должен проводиться уже на данном этапе нарушений углеводного обмена [6]. Исследование KORAF4, целью которого была оценка распространенности нейропатии у лиц при предиабете, показало, что из 46 больных нейропатия встречалась в 23,4% случаев [7]. Однако SeigoKuris и соавт., исследуя распространенность нейропатии среди населения Японии, не обнаружили поражения периферических нервов у группы пациентов с предиабетом, как и у лиц с нормальным углеводным обменом, в отличие от больных с уже установленным СД [8].

В ряде случаев диабетической нейропатии развивается характерное поражение костей и суставов, называемое диабетической нейроостеоартропатией (ДНОАП), или стопой Шарко [9]. Под ДНОАП понимают поражение костей и суставов неинфекционного генеза на фоне нарушения периферической иннервации с признаками асептического воспаления в острой фазе заболевания [10]. По данным литературы, частота ДНОАП в популяции пациентов с диабетом составляет около 0,16% [11], а у больных с диабетической нейропатией может достигать 29%. В других исследованиях частоту ДНОАП оценили в 0,08% в популяции лиц с СД и в 13% — у лиц с высоким риском диабета и периферической нейропатией [12][13]. Также данные о распространенности этого осложнения диабета зависят от метода диагностики поражения костно-суставной системы и длительности заболевания диабетом исследуемой группы. Miller D.S. и соавт., обследуя неоднородную группу больных и ориентируясь только на клинические признаки артропатии, выявили ДНОАП в 5,9% случаев [14]. В другом исследовании изучение группы лиц с длительно текущим СД 1 типа с помощью сцинтиграфии выявило двустороннее поражение костей стоп у 75% обследованных [15].

К причинам столь различных данных о частоте ДНОАП можно отнести отсутствие обширных популяционных исследований и затруднение в постановке данного диагноза, вызванное недостаточным уровнем настороженности у медицинского персонала в отношении ранних признаков заболевания. Значимую роль в частоте выявления ДНОАП играют такие факторы, как возможность раннего обращения к врачу, доступность рентгенографии и магнитно-резонансной томографии (МРТ). Также стоит отметить, что чем более специализирована клиника или центр, в которых проводятся исследования, тем выше частота диагностики данного осложнения СД [15][16].

Данные о распространенности ДНОАП варьируют в широких пределах ввиду влияния различных факторов (медицинских, организационных), которые отличаются в разных системах здравоохранения.

Несмотря на существование двух основных теорий, объясняющих патогенез стопы Шарко, полного понимания и раскрытия сути механизма развития патологических процессов при ДНОАП все еще нет [17][10].

Первая теория, нейроваскулярная, объясняет возникновение остеоартропатии активацией остеокластов и, как следствие, развитием локальной остеопении в результате патологического артериовенозного шунтирования крови через сосудистое русло костной ткани. Согласно второй теории, нейротравматической, основной механизм патологического процесса заключается в моторной нейропатии, которая вследствие слабости связочного аппарата приводит к увеличению амплитуды движений в суставах, их нестабильности и повреждению при незначительных травмах [18][19].

Помимо того, что кость теряет плотность, она становится менее эластичной по причине нарушения свойств коллагеновых волокон, в результате чего скелет стопы становится менее устойчивым к повреждениям. Данное обстоятельство приводит к тому, что даже незначительная механическая травма может стать причиной фрагментации костей, вывихов и подвывихов суставов. Дополнительное влияние на патологический процесс оказывает отсутствие болевой чувствительности, в связи с чем нагрузка на пораженную конечность не ограничивается. Это приводит к повторным повреждениям связочного аппарата, следствием чего является запуск воспалительного процесса.

Воспалительная реакция, возникающая в ответ на какое-либо повреждение, вызывает усиление лизиса кости за счет повышенной экспрессии провоспалительных цитокинов, включая IL-1β, IL-6, TNF-α [17]. Однако до сих пор неясно, является ли это причиной или следствием продолжающегося процесса костной резорбции. В исследовании, проведенном Jansen и соавт., было обнаружено, что у пациентов с ДНОАП венозно-артериальный поток IL-6 в пораженной стопе, в отличие от здоровой, увеличивается. Это позволяет предположить, что IL-6 локально продуцируется именно на месте воспаления [20].

Цитокины — это группа регуляторных пептидов, продуцируемых клетками организма. Ключевыми из них являются IL-1, IL-6, TNF, а также хемокины.

Они стимулируют развитие воспалительной реакции при внедрении патогенов и повреждение тканей, тем самым способствуя выведению чужеродного агента [21][22].

Повышение экспрессии RANKL (полипептида лиганда рецептора активатора ядерного фактора каппа-β) из остеобластов может происходить за счет провоспалительных цитокинов [23].

RANKL, связываясь с рецептором RANK на поверхности остеокласта, способствует активации факторов транскрипции (NF-kB), что, в свою очередь, приводит к образованию остеокласта из его предшественника [24].

Для поддержания баланса в процессе костного ремоделирования остеобластами вырабатывается остеопротегерин, являющийся антагонистом RANKL. Однако у пациентов с ДНОАП преобладают процессы костной резорбции, которые связаны с выявленными полиморфизмами в гене OPG, приводящие к изменению соотношения OPG/RANKL [25].

Помимо вышеперечисленных факторов, важную роль в формировании микро- и макрососудистых осложнений СД играют конечные продукты гликирования (КПГ). КПГ представляют собой различные соединения, которые образуются в результате реакции между углеводами и свободными аминогруппами белков, липидов и нуклеиновых кислот без участия ферментов [26]. В процессе образования КПГ принято выделять несколько стадий. Первый этап — образование основания Шиффа, первичного продукта реакции моносахарида с аминогруппой белка, и обратимая перестройка его в более стабильные продукты Амадори. Далее образуются карбонильные интермедиаты, которые являются промежуточными продуктами гликирования и обладают более высокой реакционной способностью с аминогруппами. Заключительный этап гликирования — образование различных по структуре КПГ из продуктов Амадори и карбонильных соединений [27].

Существуют различные факторы, которые приводят к накоплению КПГ в организме человека. Выделяют эндогенные и экзогенные факторы [28][29]. К первым относятся определенные заболевания, такие как СД, почечная недостаточность, которые ускоряют процессы гликирования. К экзогенным факторам, приводящим к образованию продуктов липоокисления и гликоокисления, относятся табачный дым и длительная термическая обработка пищи [30][31].

КПГ накапливаются в костях, хрящах, мышцах, сухожилиях, связках и нервах, где они негативно влияют на биомеханические свойства тканей. Неферментативному гликированию в большей степени подвергаются белки внеклеточного матрикса с длительным периодом полураспада, к которым относится коллаген [32][33].

Содержание коллагена в организме распределено следующим образом: до 50% всего коллагена содержится в костной ткани, остальная часть входит в состав соединительной ткани, кожи, стенок сосудов, хрящей и т.д. [34]. В зависимости от роли коллагена, которую он играет в том или ином органе, в разных тканях преобладают различные типы коллагена. На данный момент известно 19 типов, отличающихся между собой функциями, локализацией и первичной структурой пептидных цепей [35]. Фибриллы, в состав которых входят коллагены 1, 2, 3-го типов, составляют основу соединительной ткани организма (хрящи, кости, сухожилия) [34].

В волокнах коллагена образуются ферментативные и неферментативные межмолекулярные поперечные связи. Поперечные связи регулируются лизингидроксилазой и лизилоксидазой. Эти соединения могут образовываться только в определенных точках молекулы коллагена (лизин или гидроксилизин) и способствуют прочности костей при сохранении соответствующей эластичности [36].

При накоплении КПГ в волокнах коллагена происходит образование неферментативных поперечных связей белков внутриклеточного и межклеточного матрикса без участия ферментативных реакций, в результате чего коллаген становится жестким и  менее восприимчивым к  протеолитическому расщеплению [37][28].

В развитии диабетических осложнений важная роль отводится КПГ, содержание которых увеличивается в зонах повреждения сосудов органов мишеней СД. Происходит это в результате снижения интенсивности внутриклеточного протеолиза [38][39].

Достоверным фактором развития осложнений диабета является пентозидин — наиболее часто определяемый КПГ. Cодержание пентозидина в значительной степени коррелирует с общим содержанием КПГ в кости [40]. В работе, проведенной Saito и соавт., в костях крыс с СД отмечалось увеличение пентозидиновых сшивок, которое отрицательно влияло на механические свойства кости. [41]. В исследовании Schwartz и соавт. было продемонстрировано наличие связи между высоким уровнем пентозидина мочи и повышенным фактором риска переломов у пожилых пациентов с СД 2 типа [42]. Проспективное когортное исследование группы женщин в пери- и постменопаузе показало, что уровни пентозидина в моче являются значимым предиктором малотравматичного перелома позвонков [43]. У женщин в постменопаузе с СД 2 типа и переломами позвоночника наблюдалось повышение уровня пентозидина [44]. Анализ образцов, полученных при биопсии гребня подвздошной кости у пациентов с СД 1 типа, показал, что содержание пентозидина в костях у больных с переломом было значительно увеличено по сравнению с людьми без костных повреждений [45]. Стоит учитывать, что вышеуказанные состояния не являются следствием пониженной минеральной плотности кости. Хрупкость костей у пациентов с диабетом связана с повышенным уровнем неферментативно сшитых КПГ в молекулах коллагена, которые снижают прочность кости.

Метаболизм КПГ включает в себя не только их образование и выведение, но и связывание их с рецепторами RAGE (receptors for advanced glycatedend-products). При взаимодействии КПГ со своими рецепторами происходит активация различных сигнальных систем [46]. RAGE — мультилиганд трансмембранного гликопротеина 1 типа, структура которого схожа с иммуноглобулином. Он состоит из внеклеточного, внутриклеточного и трансмембранного домена. Лигандами RAGE, помимо самих КПГ, являются белки семейства S-100, β-амилоид [47].

Рецептор КПГ экспрессируется на мембранах моноцитов, фибробластов, остеобластов, нейронов и т.д. [48][49].

В процессе взаимодействия с RAGE КПГ запускают активацию вторичных мессенджеров, к которым относится протеинкиназа C. Передача сигналов RAGE нацелена на перемещение NF-κB в ядро клетки, который увеличивает транскрипцию ряда белков, в том числе молекулы межклеточной адгезии-1, Е-селектина, эндотелина-1, сосудистого эндотелиального фактора роста (VEGF) и провоспалительных цитокинов [50]. Результатом данного процесса становится эндотелиальная дисфункция [51].

Результатом взаимодействия КПГ с их рецепторами является усиление образования активных форм кислорода путем активации НАДФ. Они образуются в митохондриях и в малых количествах являются физиологичными. Однако при избыточном накоплении АФК развивается окислительный стресс, а это, в свою очередь, вызывает деструктивные процессы в клетках (повреждение мембранных структур, окисление белков, повреждение ДНК). Оксид азота, являющийся субстратом для образования одного из АФК (пероксинитрита), участвует в регуляции метаболизма костной ткани, ингибируя остеокласты и индуцируя в низких концентрациях апоптоз в клетках-предшественниках остеокластов, но стимулирует костный метаболизм в высоких концентрациях [52][53]. При сравнении плазмы крови здоровых людей с пациентами, страдающими СД с нейроостеоартропатией Шарко или без нее, было выявлено пониженное количество антиоксидантов у лиц с ДНОАП [19].

Считается, что КПГ играют важную роль в развитии сердечно-сосудистых осложнений при СД. Сосудистые стенки теряют свою эластичность и становятся жесткими за счет гликирования коллагеновых цепей в артериолах, что является причиной фиброза миокарда и, как следствие, диастолической дисфункции [54].

Липопротеины низкой плотности (ЛПНП) также подвержены гликированию. Полученные из моноцитов макрофаги поглощают гликированные ЛПНП, тем самым стимулируя образование пенистых клеток, которые, в свою очередь, участвуют в формировании атеросклеротической бляшки [55].

Мишенью для КПГ-опосредованного повреждения также являются клубочки почек. Существуют механизмы разрушения эндогенных КПГ, к которым относятся внеклеточный протеолиз, а также опосредованное КПГ-рецептором (RAGE1) внутриклеточное поглощение и деструкция макрофагами. Поглощенные макрофагами КПГ выводятся почками [55]. На фоне СД образующиеся сшитые КПГ не восприимчивы к расщеплению. В связи с этим они накапливаются в тканях и приводят к утолщению базальной мембраны клубочков почек, гломерулосклерозу и тубулоинтерстициальному фиброзу [56][57].

Метилглиоксаль, образующийся в результате гликолиза, способствует накоплению КПГ. Его синтез увеличивается при СД. Данное вещество метаболизируется ферментом глиоксалазой 1 [58]. В исследовании, проведенном Giacco и соавт., было обнаружено, что у мышей с «нокаутным» геном, кодирующим глиоксалазу 1, появлялись признаки диабетической нефропатии через 6 мес. Животные со сверхэкспрессией этого фермента, напротив, были защищены от негативных последствий гипергликемии, демонстрируя минимальные почечные изменения [59].

## ЗАКЛЮЧЕНИЕ

Диабетическая нейроостеоартропатия является серьезным осложнением СД, генез которого не окончательно изучен. Избыточное образование и накопление КПГ могут играть важную роль в патогенезе этого осложнения СД. Более подробное изучение этого вопроса позволит разработать методы предупреждения формирования костно-суставной патологии у лиц с нарушенным углеводным обменом.
